# Examination of the bovine leukocyte environment using immunogenetic biomarkers to assess immunocompetence following exposure to weaning stress

**DOI:** 10.1186/1746-6148-7-45

**Published:** 2011-08-11

**Authors:** Aran O'Loughlin, Mark McGee, Sinéad M Waters, Sean Doyle, Bernadette Earley

**Affiliations:** 1 Animal and Bioscience Research Department, Animal & Grassland Research and Innovation Centre, Teagasc, Grange, Dunsany, Co. Meath, Ireland; 2 Livestock Systems Research Department, Animal & Grassland Research and Innovation Centre, Teagasc, Grange, Dunsany, Co. Meath, Ireland; 3 Department of Biology and National Institute for Cellular Biotechnology, National University of Ireland Maynooth, Co. Kildare, Ireland

## Abstract

**Background:**

The molecular mechanisms by which stress induces the development of pathologies remains unclear, although it is recognised that one of the major factors affecting health as a consequence of stress is the involvement of the neuroendocrine system. In cattle, a number of necessary husbandry practices have been shown to activate the stress response, yet very little is known about the impact these have at the molecular level. The objectives of the study were to characterise, in male and female beef calves, the immune response to weaning stress in bovine leukocytes at the physiological and molecular levels and to assess the difference between calves weaned in the presence of the dam and those weaned and penned away from the dam.

**Results:**

Following exposure to weaning stress, total neutrophil number and neutrophil:lymphocyte (N:L) ratio increased (P < 0.01) in calves. Additionally, expression of pro-inflammatory cytokine genes, including IL-1β, IL-8, IFN-γ and TNFα, were up-regulated (P < 0.01). Furthermore, there was increased (P < 0.001) expression of the glucocorticoid receptor, GRα, the pro-apoptotic gene, Fas and the Gram-negative pattern recognition receptor, TLR4. Calves penned away from the dam post-weaning had increased (P < 0.01) neutrophil number and N:L ratio compared with calves penned next to the dam, and female calves had higher (P < 0.05) expression levels of IL-2, IL-8, IFN-γ and TNFα than male calves.

**Conclusions:**

Weaning elicits an immediate and somewhat short-lived acute stress response in the calf. The effects serve to enhance, rather than suppress, the immune response by means of a heightened inflammatory response and cellular mobilization. The earlier and more profound increase in neutrophil number and N:L ratio together with reduced lymphocyte number in calves penned away compared with calves penned near their dams post-weaning suggests that the former may be more sensitive to weaning stress. The data also show a clear effect of gender in differential gene expression in response to stress with IFN-γ having increased expression in female calves compared with male calves over the course of the study. Additionally, this study has helped to characterise the inflammatory response to stress in calves and identify a number of novel candidate biomarkers suitable for investigation in future studies of stress.

## Background

Stress has been well documented to negatively impact the immune system in both humans and animals [1-8]. In cattle, research measuring stress-related immune function has focused on a number of husbandry management practices including castration [9-17], housing [18-21], transport [22-29], and weaning [30-35]. Weaning involves separation of the calf from its dam, resulting in a breaking of the maternal-offspring bond and removal of milk from its diet. Additionally, the calf may be exposed to other stressors including social reorganisation, housing, transport and novel handling. Thus, the weaning procedure can be an acute stressful event in young calves combining social, physical, nutritional and psychological stresses [34,35]. Hickey et al. [30] found alterations in immune function and hormonal mediators of stress still present at 7 days following abrupt weaning whereby the cows were suddenly removed from the calves. Compared with abrupt weaning, practices such as progressive weaning or fence-line weaning have reduced the frequency of behavioural distress, heart-rate and neutrophil: lymphocyte (N:L) ratio in calves [36-38]. Similarly, Lynch et al. [35] reported that deferring housing at the time of weaning resulted in a less marked stress response in beef calves compared with the traditional practice of weaning and simultaneous housing indoors at the end of the grazing season. In view of these findings, the present study was designed to remove the effect of housing stress and adaptation to a new diet at the time of weaning by housing the cows and calves 28 days prior to weaning, thus allowing the calves time to adjust to their new environment before this event. This provided the opportunity to examine both the effect of breaking the maternal-offspring bond and the effect of social reorganisation by using a fence-line weaning system [36-39] modified for a housing environment to detect if animals weaned in the presence of their dam would be less susceptible to weaning induced stress compared with those weaned and penned away from their dam. Both male and female animals were used in order to establish if physiological and molecular differences exist in their responses to stress as the literature is equivocal on this point.

To the authors' knowledge, no research has examined the molecular mechanisms underlying weaning stress, particularly with a focus on immunogenetic markers of stress. Through the use of real-time (RT)-qPCR, the expression of a number of cytokines and other key immune biomarkers of stress was used to characterise the molecular response of the calf to stress. Although bovine leukocytes are well-characterised in relation to stress [40], recent molecular studies have focused on specific cell populations [25,27] leaving leukocytes and their cytokine networks largely uncharacterised at the molecular level [41]. Examining combined leukocyte populations provides a broader picture of immunological interactions than can be garnered from focusing on particular cell types. The study hypothesis was that calves that were weaned and penned away from their dams would be more stressed than calves that were weaned and penned adjacent to their dams. Therefore, the objectives of this study were to characterise, in male and female calves, the immune response to weaning stress at the physiological and molecular levels, and to assess the difference between calves weaned and penned in the presence of the dam and those weaned and penned away from the dam.

## Methods

All animal procedures performed in this study were conducted under experimental licence from the Irish Department of Health and Children in accordance with the Cruelty to Animals Act 1876 and the European Communities (Amendment of Cruelty to Animals Act 1876) Regulation 2002 and 2005.

### Animal management

Calves were born at the Research Centre and as part of standard husbandry and research management practices were accustomed to routine handling in the facilities and to stockpersons. Twenty-eight clinically healthy, three-quarter bred Simmental beef calves were housed indoors in concrete slatted floor pens with their dams on day (d) -28 of the study. Calves were immunized on d -28 against bovine respiratory syncytial virus (BRSV) and infectious bovine rhinotracheitis (IBR) virus using *Rispoval-3 *and *Rispoval-IBR *vaccines, respectively. The calves were offered a new diet of grass silage (mean dry matter digestibility (DMD) (s.d.) 719 (16.3) g/kg; mean crude protein (CP) concentration (s.d.) 145 (11.8) g/kg dry matter (DM)) and had free access to concentrates (931 g/kg barley, 50 g/kg molasses and 15 g/kg minerals and vitamins (mean DMD (s.d.) 825 (7.9) g/kg, mean CP (s.d.) 108 (9.2) g/kg DM, mean neutral detergent fibre (NDF) (s.d.) 188 (19.6) g/kg DM)) per animal daily. Feedstuff analysis was determined as described by Owens et al. [42]. At weaning (d 0), the cows were removed and the calves were regrouped and assigned to new pens with concrete slatted floors (mean space allowance of 2.9 m^2^/head/calf) mimicking social reorganisation, and allocated to one of four treatments: 1), females weaned beside the dam (n = 7; mean weight (s.d.) 267.3 (45.1); mean age (s.d.) 227 (17.9) d), 2), females weaned away from dam (n = 7; mean weight (s.d.) 274.1 (35.3) kg; mean age (s.d.) 224 (25.1) d), 3), males weaned beside the dam (n = 7; mean weight (s.d.) 270.6 (69.8) kg; mean age (s.d.) 197 (38.1) d), or 4), males weaned away from dam (n = 7; mean weight (s.d.) 267.9 (82.8) kg; mean age (s.d.) 209 (37.3) d). Treatments 1 and 3 were assigned to pens adjacent to their dam. A metal grid fence prevented suckling whereas visual and auditory contact was allowed. Treatments 2 and 4 were assigned to pens sufficiently distant from their dam to prevent any visual or auditory contact.

### Environmental measurements

Shed and ambient temperatures were monitored using data loggers (Testo 175 data loggers, Eurolec, Ireland).

### Rectal temperature measurements

Rectal body temperature was recorded while the calves were waiting in the holding chute immediately prior to blood sample collection using a digital thermometer (Jorgen Kruuse A/S; Model VT-801 BWC Lot No 0701, Marslev, Denmark).

### Blood sample collection

Calves were blood sampled via jugular venipuncture on days (d) -3, 0, 1, 2, 3, 7, and 11 relative to weaning (d 0). For this procedure, the calves were led gently to a holding pen, with a squeeze chute facility and were blood sampled with minimal restraint. Blood sampling was carried out by the same experienced operator on each occasion and the time taken to collect the blood samples was less than 60 s/calf. Blood samples were collected into 1 × 6 mL K_3_Ethylenediaminetetraacetic acid (K_3_EDTA) tubes (Vacuette, Cruinn Diagnostics, Ireland) for haematological analysis and into 5 × 9 mL acid citrate dextrose (ACD) tubes (Vacuette, Cruinn Diagnostics, Ireland) for leukocyte isolations.

### Haematology

Unclotted whole K_3_EDTA blood samples were analysed using an ADVIA haematology analyser (AV ADVIA 2120, Bayer Healthcare, Siemens, UK) equipped with software for bovine blood. Total leukocyte, neutrophil, lymphocyte, eosinophil and monocyte number, red blood cell (RBC) number, haemoglobin (HGB), mean corpuscular haemoglobin concentration (MCHC), haematocrit (HCT) percentage and platelet (PLT) number were measured. The N:L ratio was also calculated.

### Leukocyte isolation from whole blood

Thirty-six mL of blood from the ACD tubes was pooled from each animal within 30 minutes of collection and split into three 12 mL aliquots. Red blood cells were lysed for 90 seconds using 24 mL of a hypotonic solution (10 mM Na_2_HPO_4_, 2 mM NaH_2_PO_4; _pH 7.2) followed by restoration using 12 mL of a hypertonic solution (10 mM Na_2_HPO_4_, 2 mM NaH_2_PO_4_, 461 mM NaCl; pH 7.2). The tubes were then centrifuged at 1800 rpm for 5 minutes at 4°C to collect the leukocyte pellet. The leukocyte pellet was washed twice by resuspending in Dulbecco's phosphate buffered saline (DPBS) and centrifuged for 5 minutes. The isolated leukocytes were resuspended in 1 mL of TRI Reagent (Sigma-Aldrich Ireland Ltd., Dublin, Ireland), pooled by animal and stored in a sterile tube at -80°C until RNA extractions were performed.

### RNA Extraction and cDNA Synthesis

A modified TRI Reagent extraction method [43] was used to extract total RNA from leukocytes via homogenization of the pellet in TRI Reagent and the subsequent addition of chloroform followed by precipitation using isopropanol and ethanol. RNA was quantified using a Nanodrop Spectrophotometer (NanoDrop Technologies, Wilmington, DE, USA). RNA quality was assessed on an Agilent 2100 Bioanalyser (Agilent Technologies Ireland Ltd., Dublin, Ireland) and only RNA samples with a RNA Integrity Number (RIN) of greater than 8.0 were used. Samples were DNase treated and purified using an RNeasy mini kit (Qiagen Ltd., Crawley, UK). One μg of total RNA per animal was reverse transcribed into cDNA using random hexamers and the High Capacity cDNA Reverse Transcription kit (Applied Biosystems, Ireland) in a 20 μl reaction and stored at -20°C.

### Real-Time (RT)-qPCR

Primers for candidate genes (Table 1) were designed based on bovine sequences obtained from the NCBI database using Primer3 software [44] and were commercially synthesised (Sigma-Aldrich Ireland Ltd., Dublin, Ireland). Amplified PCR products were sequenced (Cambridge, UK) and verified with BLAST http://blast.ncbi.nlm.nih.gov/ to be identical to their respective bovine sequence. Amplification efficiencies were determined for all genes using serial dilutions of pooled cDNA samples. The formula E = -1 + 10^(-1/slope) ^was used where slope refers to the slope of the linear curve of cycle threshold (Cq) values plotted against log dilution [45]. Only primers with PCR efficiencies between 90 and 110% were used in the current study. Reference genes used in this study were selected using the geNorm software v3.5 [46]. Based on the average pairwise variation, V, three genes (beta-actin (*ACTB*), succinate dehydrogenase complex subunit A (*SDHA*) and glyceraldehyde 3-phosphate dehydrogenase (*GAPDH*)), were found to have an average stability value of M = 0.24. A normalisation factor, calculated based on the geometric mean of the three reference genes, was used to normalise the expression of each gene of interest.

**Table 1:** Primers for RT-qPCR candidate genes were designed using Primer3 software [44] and based on bovine sequences obtained from the NCBI database.

Gene		Sequence 5' → 3'	Amplicon Size	NCBI Accession
IL1β	F:	CAGTGCCTACGCACATGTCT	167 bp	NM_174093
	R:	CCAGGGATTTTTGCTCTCTG		
IL2	F:	GTGAAGTCATTGCTGCTGGA	101 bp	NM_180997
	R:	GGCGCGTAAAAGTCAAATGT		
IL4	F:	CTGCCCCAAAGAACACAACT	165 bp	EU276069
	R:	TCGTCTTGGCTTCATTCACA		
IL8	F:	TGGGCCACACTGTGAAAATTC	92 bp	[16]
	R:	CCTTCTGCACCCACTTTTCC		
IFNγ	F:	TTCAGAGCCAAATTGTCTCC	205 bp	[89]
	R:	AGTTCATTTATGGCTTTGCGC		
TNFα	F:	TGGAGGGAGAAGGGATTCTT	140 bp	AF011926
	R:	CCAGGAACTCGCTGAAACTC		
Lymphotoxin	F:	GCTGCATCCCTAAGAACAGC	141 bp	BC149732
	R:	CATCCGGCTCAAAAATCAGT		
TLR4	F:	TGGTAAACCCCAGAGTCCAG	164 bp	NM_174198
	R:	GCACAATGCTTGGTACATGG		
NFkB1	F:	GCACCACTTATGACGGGACT	148 bp	NM_001076409
	R:	TCCTCATCCCAGGAGTCATC		
NFkB2	F:	ATCTGAGCATTGTGCGACTG	131 bp	NM_001102101
	R:	CTTCAGGTTTGAGGCTCCAG		
GRα	F:	CCATTTCTGTTCACGGTGTG	132 bp	AY238475
	R:	CTGAACCGACAGGAATTGGT		
Fas	F:	AGTTGGGGAGATGAATGCTG	171 bp	NM_174662
	R:	CCTGTGGATAGGCATGTGTG		
Haptoglobin	F:	TGGTCTCCCAGCATAACCTC	185 bp	BC109668
	R:	AGGGTGGAGAACCACCTTCT		
CD62L	F:	CCGATTGCTGGACTTACCAT	194 bp	NM_174182
	R:	CCAAGTCCACACCCCTTCTA		
p21	F:	TCCAAGGACTTTTTCCATTTGC	75 bp	[25]
	R:	TCTGACTCCTTCAGCTGTTATTCAA		
BPI	F:	TTCAGAAATGATCCAAACATGAAAC	81 bp	[25]
	R:	GCCCTTGGAAGAAACAATTCC		
ACTB	F:	ACTTGCGCAGAAAACGAGAT	123 bp	BT030480
	R:	CACCTTCACCGTTCCAGTTT		
SDHA	F:	AACTGCGACTCAACATGCAG	132 bp	NM_001034034
	R:	TGTCGAACGTCTTCAGATGC		
GAPDH	F:	GGGTCATCATCTCTGCACCT	176 bp	DQ402990
	R:	GGTCATAAGTCCCTCCACGA		

Real-time RT-qPCR was performed using the Applied Biosystems 7500 FAST RT-PCR equipment v2.0.1 (Applied Biosystems, Ireland). One μL of cDNA was added to a 19 μL master mix which included 10 μL of Fast SYBR Green I master mix (Applied Biosystems, Ireland), 8 μL of nuclease-free water and 0.5 μL each of forward and reverse primers at concentrations individually optimised for each primer set. The following RT-qPCR cycle conditions were applied: 95°C for 20 s followed by 40 cycles of 95°C for 3 s and 60°C for 30 s, finishing with amplicon dissociation at 95°C for 15 s, 60°C for 1 min increasing 0.5°C per cycle until 95°C was reached for 15 s followed by 60°C for 15 s.

In accordance with the MIQE guidelines [47,48], raw Cq values were imported to GenEx Software v.5.2.2.8 (2010) (MultiD Analyses AB, Göteborg, Sweden). Outliers were removed from replicate wells using a modified Grubbs test [49,50] at a P < 0.05 confidence interval for any replicate differing from the replicate mean by a standard deviation of more than 0.25 cycles. Adjustments were made for interplate variation based on calibrator samples included on all plates. The Cq values were adjusted for calculated efficiencies before averaging of replicates. These values were then normalised to the reference genes followed by calculation of relative quantities to the highest Cq value.

### Statistical analyses

All data were checked for adherence to normal distributions (PROC UNIVARIATE, SAS v 9.1). The natural logarithm transformation was used to normalize the distributions of neutrophil and eosinophil numbers. Interleukin (IL) -1β, IL-2, IL-4, IL-8, interferon (IFN) -γ, tumour necrosis factor (TNF) -α, lymphotoxin, toll-like receptor (TLR) 4, glucocorticoid receptor (GR) α, nuclear factor (NF) κB1, NFκB2, Fas, p21, CD62L, haptoglobin, bactericidal/permeability increasing protein (BPI)) were log_2 _transformed (GenEx Software v.5.2.2.8 (2010) (MultiD Analyses AB, Göteborg, Sweden)) prior to statistical analysis. Haematological data, relative gene expression and rectal temperature data were analysed as a 2 × 2 factorial with repeated measures using the PROC MIXED procedure of SAS (Version 9.1, SAS Institute, Cary, NC). The effects of treatment, sampling time, gender and all possible interactions were listed in the model statement. Animal was the experimental unit and was specified as a repeated measures effect, and the dependence within animal was modelled using an unstructured covariance structure. For haematological data and rectal temperature measurements, the first sample (d -3; sample 1) was used as the baseline covariate in the statistical analysis of the data. Differences between means were tested using the PDIFF option in PROC MIXED of SAS. Means were considered significantly different at the P < 0.05 probability level.

## Results

### Environmental measures

The temperature of the housing environment ranged from 3.9°C to 17.4°C with a mean (s.d.) temperature of 9.8°C (2.2). The ambient temperature ranged from 2.8°C to 26.0°C with a mean (s.d.) temperature of 9.2°C (3.4).

### Rectal body temperature

Rectal body temperature was higher (P < 0.01) for away calves than near calves (data not shown). There was a gender × time (P < 0.01) interaction whereby rectal temperature was lower than baseline on d 2, 3 and 11, particularly in female calves.

### Leukocyte population

**Figure 1:**
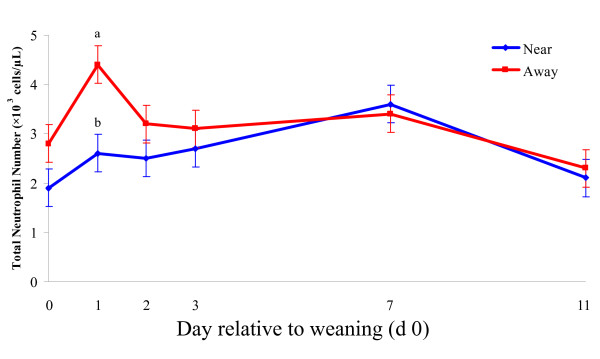
**Alterations in total neutrophil number between calves weaned in the presence of the dam and those weaned and penned away from the dam**. Neutrophil number for calves weaned away from the dam was significantly higher on day 1 than for calves weaned beside the dam, indicating an effect of location. ^a, b^Lsmeans differ between location by *P *< 0.05.

There was no effect of gender, location and time or their interaction on total leukocyte or monocyte number (Table 2). There was a location × time interaction (P < 0.01) but no effect (P > 0.05) of gender for total neutrophil number (Table 2; Figure 1). Following weaning, neutrophil number increased in all calves (P < 0.001) but the increase was greater (P < 0.001) in calves weaned away from the dam than those weaned beside the dam. Neutrophil number returned to baseline by d 11. Lymphocyte number decreased (P < 0.05) from baseline on d 2 and remained lower throughout the study. There was a gender × location interaction (P < 0.01) for lymphocyte number, whereby away males had lower (P < 0.05) lymphocyte numbers than near males and the opposite (P < 0.01) occurred with females. There was a location × time (P < 0.01) interaction for N:L ratio (Table 2). Post-weaning N:L ratio increased (P < 0.01) from baseline and did not return to pre-weaning levels until d 11, but on d 1, the increase was greater (P < 0.01) in away calves than in near calves. There was no effect (P > 0.05) of gender on N:L ratio. A gender × time effect (P < 0.01) existed for eosinophil number (Table 2) whereby eosinophil number in males decreased (P < 0.01) from baseline on d 1 and decreased (P < 0.01) in females on d 7.

**Table 2:** Effect of weaning induced stress on total leukocyte, neutrophil and lymphocyte number, neutrophil:lymphocyte (N:L) ratio, eosinophil and monocyte number in weaned beef calves.

	**Gender**		**Location**		**s.e**	**Time**						**s.e**.	***P*-Values**			
				
**Variable**	**Male**	**Female**	**Near**	**Away**		**0**	**1**	**2**	**3**	**7**	**11**		**G**	**L**	**T**	**I**
				
**Total Leukocytes**	9.9	9.7	9.4	10.2	0.40	9.8	10.5	9.8	9.7	10.5	8.8	0.41	NS	NS	NS	NS
(×10^3 ^cells/μL)																
**Neutrophils**	3.1	2.6	2.5	3.2	0.29	2.3	3.5^c^	2.9^b^	2.9^b^	3.5^c^	2.2	0.27	NS	NS	***	**^1^
(×10^3 ^cells/μL)																
**Lymphocytes**	7.1	6.7	6.8	6.9	0.13	7.2	7.2	6.8^b^	6.8^b^	6.9^a^	6.5^c^	0.13	*	NS	*	**^2^
(×10^3 ^cells/μL)																
**N:L**	0.46	0.38	0.39	0.45	0.04	0.33	0.49^c^	0.43^b^	0.42^b^	0.52^c^	0.33	0.04	NS	NS	**	**^3^
(Ratio)																
**Eosinophils**	0.12	0.13	0.12	0.13	0.01	0.13	0.10^a^	0.11	0.15	0.13	0.10^b^	0.01	NS	NS	NS	**^4^
(×10^3 ^cells/μL)																
**Monocytes**	0.59	0.57	0.55	0.61	0.03	0.60	0.54	0.62	0.58	0.58	0.57	0.03	NS	NS	NS	NS
(×10^3 ^cells/μL)																

The values are expressed as least squares means (Lsmeans) and s.e.

G = gender, L = location, T = time, I = interaction, NS = not significant (*P *> 0.05). * = *P *< 0.05, ** = *P *< 0.01, *** = *P *< 0.001.

^a, b, c^Within rows, Lsmeans differ from pre-weaning baseline by *P *< 0.05, *P *< 0.01 and *P *< 0.001, respectively.

^1^Location × Time interaction, 1.9 *vs *2.8, 2.6 *vs *4.4, 2.5 *vs *3.2, 2.7 *vs *3.1, 3.6 *vs *3.4, 2.1 *vs *2.3 for near and away calves at time 0, 1, 2, 3, 7 and 11, respectively.

^2^Gender × Location interaction, 6.9 *vs *6.4 and 6.7 *vs *7.5 for near and away male and female, respectively; away male 6.4 *vs *away female 7.5.

^3^Location × Time interaction, 0.27 *vs *0.38, 0.38 *vs *0.59, 0.40 *vs *0.45, 0.41 *vs *0.42, 0.54 *vs *0.48, 0.33 *vs *0.33 for near and away calves at time 0, 1, 2, 3, 7 and 11, respectively.

^4^Gender × Time interaction, 0.12 *vs *0.15, 0.12 *vs *0.08, 0.14 *vs *0.10, 0.17 *vs *0.12, 0.13 *vs *0.14, 0.08 *vs *0.12 for male and female calves at time 0, 1, 2, 3, 7 and 11, respectively.

### Red blood cell number, haemoglobin concentration, haematocrit percentage, mean corpuscular haemoglobin concentration and platelet number

There was no effect (P > 0.05) of gender, location and time or their interaction on HGB concentration (Table 3). Number of RBC (P < 0.001) and HCT % decreased (P < 0.01) from baseline on d 11 post weaning. There was a location × time × gender interaction (P < 0.05) for MCHC. MCHC increased (P < 0.05) from baseline on d 1 in away calves and on d 2 in female calves, while increasing (P < 0.001) from baseline substantially in all animals on d 11, and was higher (P < 0.05) in away animals on d 2. There was a gender × time interaction (P < 0.01) for platelet number, whereby males had higher (P < 0.01) platelet numbers than females in the first 3 days following weaning, before decreasing (P < 0.001) from baseline in all calves on d 11. However, there was no effect (P > 0.05) of location on platelet number.

**Table 3:** Effect of weaning induced stress on red blood cell (RBC) number, haemoglobin (HGB) concentration, haematocrit (HCT) %, mean cell haemoglobin concentration (MCHC) and platelet number in weaned beef calves.

	**Gender**		**Location**		**s.e**	**Time**						**s.e**.	***P*-Values**			
				
**Variable**	**Male**	**Female**	**Near**	**Away**		**0**	**1**	**2**	**3**	**7**	**11**		**G**	**L**	**T**	**I**
				
**RBC**	10.2	9.5	9.8	9.9	0.29	10.6	10.6	10.2	10.7	10.7	6.6^c^	0.24	NS	NS	***	NS
(×10^6 ^cells/μL)																
**HGB**	12.4	12.3	12.4	12.3	0.10	12.4	12.5	12.4	12.6	12.2	12.2	0.10	NS	NS	NS	NS
(g/dL)																
**HCT**	31.6	30.9	31.5	31.0	0.67	33.7	33.5	32.4^a^	33.9	33.1	21.1^c^	0.62	NS	NS	**	NS
(%)																
**MCHC**	41.5	40.9	40.8	41.6	0.59	36.9	37.4	38.2	36.9	36.9	60.7^c^	0.93	NS	NS	***	*^1^
(g/dL)																
**Platelet**	872.1	741.1	819.0	794.1	31.6	815.9	952.5^c^	953.2^c^	882.8	739.5^a^	495.6^c^	31.8	**	NS	**	**^2^
(×10^3 ^cells/μL)																

The values are expressed as least squares means (Lsmeans) and s.e.

G = gender, L = location, T = time, I = interaction, NS = not significant (*P *> 0.05). * = *P *< 0.05, ** = *P *< 0.01, *** = *P *< 0.001.

^a, b, c^Within rows, Lsmeans differ from pre-weaning baseline by *P *< 0.05, *P *< 0.01 and *P *< 0.001, respectively.

^1^Gender × Location × Time interaction, near male 57.9 *vs *away male 68.1 at time 7

^2^Gender × Time interaction, 857.9 *vs *773.8, 1043.4 *vs *861.7, 1037.9 *vs *868.5, 1013.9 *vs *751.7, 787.8 *vs *691.2, 491.6 *vs *499.7 for male and female calves at time 0, 1, 2, 3, 7 and 11, respectively.

### Cytokine gene expression

**Figure 2:**
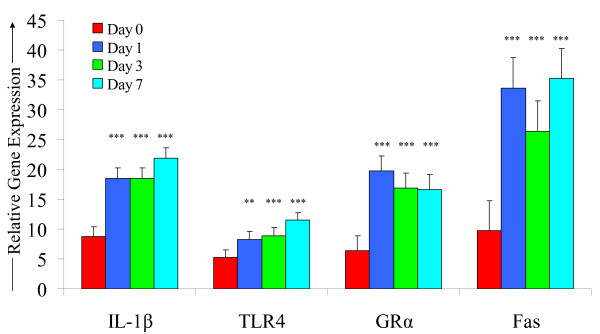
**The effect of weaning induced stress on the relative gene expression of IL-1β, TLR4, GRα and Fas**. These profiles demonstrate a clear effect of weaning stress on the expression of a number of genes. However, no effect of location was detected. * = *P *< 0.05, ** = *P *< 0.01, *** = *P *< 0.001. Lsmeans differ relative to pre-weaning baseline.

**Figure 3:**
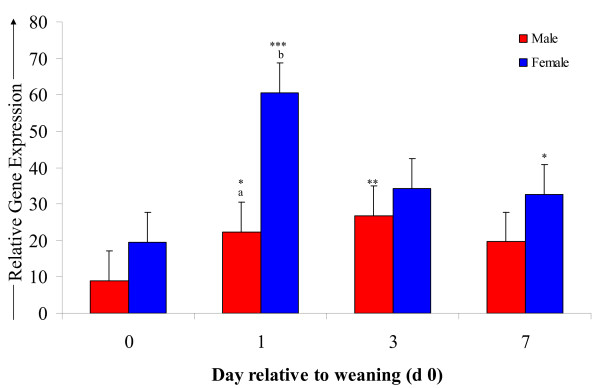
**The effect of weaning induced stress on the relative gene expression of IFN-γ**. A clear effect of gender can be seen whereby female calves had increased expression of IFN-γ versus male calves following weaning. * = *P *< 0.05, ** = *P *< 0.01, *** = *P *< 0.001. Lsmeans differ relative to pre-weaning baseline. ^a, b^Lsmeans differ between gender by *P *< 0.05.

There was no effect (P > 0.05) of gender, location and time or their interaction on expression of IL-4 and lymphotoxin, and no effect (P > 0.05) of location on IL-1β, IL-2, IL-8, IFN-γ and TNFα (Table 4). Expression of IL-1β increased over 2-fold (P < 0.001) from baseline on d 1 following weaning and remained increased on d 3 and 7 (Table 4; Figure 2). There was a gender × time interaction (P < 0.05) for expression of IL-2 (Table 4), whereby an increase (P < 0.05) from baseline occurred in females on d 1 but not in males. Female calves had greater (P < 0.05) expression of IL-8 versus male calves. Expression of IL-8 increased more than 2-fold (P < 0.001) from baseline on d 1 and remained elevated (P < 0.05) on d 3 (Table 4). There was a gender × time interaction (P < 0.05) for IFN-γ (Table 4; Figure 3) and TNFα (Table 4) gene expression. The mRNA expression of IFN-γ increased over 3-fold (P < 0.001) from baseline in all calves on d 1 and remained elevated (P < 0.01) on d 3 and d 7. IFN-γ expression was significantly (P < 0.001) higher in female calves on d 1 versus male calves. TNFα expression increased (P < 0.001) from pre-weaning baseline on d 1 and remained elevated (P < 0.01) on d 3 and d 7. However, expression levels of TNFα were significantly greater in females on d 1 (P < 0.01) and d 7 (P < 0.05) compared with males.

**Table 4:** Effect of weaning induced stress on the relative gene expression of IL-1ß, IL-2, IL-4, IL-8, IFN-γ, TNFα and lymphotoxin in weaned beef calves.

	**Gender**		**Location**		**s.e**.	**Time**				**s.e**.	***P*-Values**			
				
**Variable**	**Male**	**Female**	**Near**	**Away**		**0**	**1**	**3**	**7**		**G**	**L**	**T**	**I**
				
**IL-1β**	17.7	16.2	16.6	17.3	1.3	8.7	18.5^c^	18.5^c^	21.9^c^	1.7	NS	NS	***	NS
**IL-2**	9.9	10.8	11.8	8.9	1.9	9.6	12.6	10.1	9.2	1.8	NS	NS	NS	*^1^
**IL-4**	11.8	11.9	13.6	10.1	1.8	11.5	15.3	10.5	10.2	1.8	NS	NS	NS	NS
**IL-8**	7.1	11.5	8.9	9.7	1.3	5.6	12.9^c^	9.2^a^	9.7	1.8	*	NS	**	NS
**IFN-γ**	19.4	36.8	29.3	26.8	5.8	14.2	41.4^c^	30.5^b^	26.2^b^	5.7	*	NS	***	*^2^
**TNFα**	3.0	3.7	3.5	3.3	0.22	2.4	3.8^c^	3.4^b^	3.8^c^	0.29	*	NS	***	*^3^
**Lymphotoxin**	78.6	104.1	100.3	82.4	11.9	96.1	100.6	82.1	86.5	10.2	NS	NS	NS	NS

The values are expressed as least squares means (Lsmeans) and s.e.

G = gender, L = location, T = time, I = interaction, NS = not significant (*P *> 0.05). * = *P *< 0.05, ** = *P *< 0.01, *** = *P *< 0.001.

^a, b, c^Within rows, Lsmeans differ from pre-weaning baseline by *P *< 0.05, *P *< 0.01 and *P *< 0.001, respectively.

^1^Gender × Time interaction, 9.3 *vs *9.8, 10.9 *vs *14.4, 11.8 *vs *8.4, 7.6 *vs *10.8 for male and female calves at time 0, 1, 3 and 7, respectively.

^2^Gender × Time interaction, 9.0 *vs *19.4, 22.2 *vs *60.6, 26.7 *vs *34.3, 19.6 *vs *31.7 for male and female calves at time 0, 1, 3 and 7, respectively.

^3^Gender × Time interaction, 2.4 *vs *2.4, 3.0 *vs *4.6, 3.5 *vs *3.2, 3.1 *vs *4.4 for male and female calves at time 0, 1, 3 and 7, respectively.

### Immunological biomarker gene expression

There was no effect (P > 0.05) of gender, location and time or their interaction on expression of NFκB2, p21 and haptaglobin, and no effect (P > 0.05) of gender and location or their interaction on TLR4, GRα, CD62L and Fas (Table 5; Figure 2).

**Table 5:** Effect of weaning induced stress on the relative gene expression of toll-like receptor (TLR) 4, glucocorticoid receptor (GR)α, nuclear factor kappa B (NFκB), Fas, p21, CD62l, haptoglobin and bactericidal/permeability increasing protein (BPI) in weaned beef calves.

	**Gender**		**Location**		**s.e**.	**Time**				**s.e**.	***P*-Values**			
				
**Variable**	**Male**	**Female**	**Near**	**Away**		**0**	**1**	**3**	**7**		**G**	**L**	**T**	**I**
				
**TLR4**	9.4	7.5	8.0	8.9	1.4	5.2	8.3^b^	8.9^c^	11.5^c^	1.3	NS	NS	***	NS
**GRα**	13.4	16.5	16.4	13.5	2.1	6.4	19.8^c^	16.9^c^	16.6^c^	2.5	NS	NS	***	NS
**NFκB1**	2.9	2.9	2.8	3.0	0.27	2.5	3.1^b^	2.9	3.1^b^	0.25	NS	NS	*	NS
**NFκB2**	2.9	2.8	2.9	2.7	0.22	2.8	2.9	2.7	2.8	0.24	NS	NS	NS	NS
**Fas**	27.9	24.5	25.7	26.8	4.8	9.7	33.6^c^	26.4^c^	35.2^c^	5.1	NS	NS	***	NS
**p21**	5.8	5.5	5.4	5.9	0.70	6.2	4.9	5.7	5.6	0.71	NS	NS	NS	NS
**CD62L**	33.7	35.5	34.4	34.8	5.1	26.0	34.8^b^	37.5^c^	40.1^c^	4.3	NS	NS	***	NS
**Haptoglobin**	14.4	16.6	13.7	17.3	3.9	12.8	16.3	17.7	15.2	3.5	NS	NS	NS	NS
**BPI**	14.8	15.6	16.5	13.9	2.5	18.3	16.7	14.8^a^	11.1^c^	2.1	NS	NS	***	NS

The values are expressed as least squares means (Lsmeans) and s.e.

G = gender, L = location, T = time, I = interaction, NS = not significant (*P *> 0.05). * = *P *< 0.05, ** = *P *< 0.01, *** = *P *< 0.001.

^a, b, c^Within rows, Lsmeans differ from pre-weaning baseline by *P *< 0.05, *P *< 0.01 and *P *< 0.001, respectively.

There was a significant effect of time on expression of TLR4, GRα, CD62L and Fas whereby gene expression increased on d 1 and remained elevated through to d 7 (P < 0.001). The magnitude of this expression on d 7 was over 1.5-fold for CD62L, over 2-fold for TLR4, and over 3-fold for GRα and Fas. Expression of NFκB1 increased (P < 0.05) from baseline on d 1 and d 7 post weaning. Expression of BPI (Table 5) was lower (P < 0.05) on d 3 and d 7, post weaning.

## Discussion

This study utilised a model that was designed with the objective of isolating the maternal-offspring separation and social reorganisation occurring at weaning time, in order to characterise the weaning response without the associated stress resulting from simultaneous housing and novel handling. Management factors that serve to increase the stress load on young animals result in decreased feed intake, partitioning of nutrients away from growth and immune dysfunction [51]. If left unchecked, these management factors can result in chronic stress and suppression of the immune system [52]. For this study, RT-qPCR analyses together with haematological profiles were examined to compare gene expression in blood leukocytes of weaned calves over a period of 7 days. To the authors' knowledge, this is the first study in which gene expression was measured in beef calves to assess immunocompetence following exposure to weaning stress.

Total leukocyte number did not change throughout the study which may be due to the simultaneous fluxes in individual cell types that often occurs during the physiological process to maintain homeostasis [53]. Lynch et al. [34] found increased leukocyte number in animals simultaneously housed and weaned, indicating that the failure to identify increased leukocyte number in the current study suggests a decreased stress load by separating housing and weaning into two distinct stages.

The significant increase in neutrophil number observed within 24 hours followed by a return to baseline by day 11 in this study is in agreement with the findings of Hickey et al. [30], Blanco et al. [32] and Lynch et al. [34] who reported increased neutrophil percentage following weaning. In the present study, neutrophilia was greater in away animals, particularly males, suggesting the lack of contact with the dam following weaning may increase the stress load in calves. Hickey et al. [30] reached similar conclusions, finding increased neutrophil number in bull calves following abrupt weaning and suggesting that male calves experience a greater difficulty dealing with the stress of weaning than female calves as plasma noradrenaline also remained elevated 7 days following weaning in males. An initial surge in the endogenous glucocorticoid, cortisol, following weaning may have increased the number of circulating neutrophils via the decreased expression of the surface marker CD62L, preventing margination and subsequent migration from the vasculature [54] along with a later burst of mature cells from the bone marrow [53].

In the present study, lymphocyte number decreased on day 2, a finding that concurs with a number of previous weaning studies [30,32,34]. This reduction in lymphocyte number may be attributed to the trafficking of lymphocytes from general circulation into tissues and organs at risk of infection [55]. Differences in lymphocyte distribution resulting from gender may not be surprising as it has previously been reported that gender differences exist in response to stress [30,56]. However, a pen effect may also exist by which the pen and not the gender of the animals may account for the differences identified between male and female calves. To control for this, in the present study, all animal pens, housing the male and female calves, were located adjacent to each other and were of similar dimension and orientation in the housing shed. Further research may be required to elucidate the full extent of gender differences. The N:L ratio is often viewed as a sensitive measurement of the relationship between neutrophils and lymphocytes, accounting for bidirectional alterations in cellular numbers and acts as an accurate indicator of stress [57]. The increase in N:L ratio during the first 24 h was more profound in the away calves than the near calves, which suggests that calves experienced a heightened stress response when they were penned away from their dam. By day 11, N:L ratio had returned to baseline levels, indicating the animals had become adapted to their penning environment. Hickey et al. [30] and Blanco et al. [32] reported that N:L ratio returned to pre-weaning baseline by day 7 post-weaning. While monocytes are generally regarded as an accurate biomarker of stress [8,55], this is not the case in the bovine due to the relative variability of monocyte distribution in cattle and their lack of sensitivity to stress hormones [53]. This is verified by the findings of this study in which no major changes were detected in monocyte number although it is important to note that monocyte biological function remains important.

Dhabhar et al. [58] examined the effect of stress-induced changes in leukocyte number and concluded that stress-induced alterations in cellular populations were rapidly reversed (minutes to hours) following elimination of the stressor. Given the aforementioned results, this suggests that weaning results in a relatively short lived (less than 11 days) acute stressor and reestablishment of cellular homeostasis by 11 days post weaning indicates an adjustment by the calves to post weaning conditions.

In the present study, the relative gene expression of a number of pro- (IL-1β, IL-2, IL-8, IFN-γ, TNF-α and Lymphotoxin) and anti- (IL-4) inflammatory cytokines was measured in order to ascertain if an inflammatory stress response was evident following weaning. As IL-8 is a well recognised neutrophil chemoattractant, it was expected that mRNA expression would increase following weaning. The over 2-fold increase in IL-8 expression on day 1 may be responsible for the reported neutrophilia. IL-8 is expressed early in the inflammatory response by macrophages at the site of inflammation and not in general circulation, suggesting transcript abundance may have already peaked by the initial post-weaning sampling time at 24 hours. Buckham Sporer et al. [25] found increased expression of IL-8 at 4.5 hours following the onset of transport and this had returned to baseline levels by 24 hours. It is probable that if more frequent blood samples were collected in the first 24 h post weaning that greater mRNA abundance of IL-8 may have been detected.

A relationship exists between the pro-inflammatory antiviral cytokine IFN-γ and the anti-inflammatory mediator IL-4 whereby the secretion of one will dictate T helper (Th) cell differentiation and abrogate expression of the other [59]. Transcript abundance of IFN-γ increased considerably following weaning in the current study, promoting a cell-mediated inflammatory response while the expression of IL-4 did not increase significantly. Morinobu and Kumagai [59] found that increased expression of IFN-γ promotes cell-mediated immunity whereas increased IL-4 promotes the humoral immune response. Therefore, the IFN-γ secretion by Th1 cells in this study skewed the immune response by increasing innate cell-mediated immunity, activating neutrophils and macrophages [60], stimulating CD4+ cell differentiation towards Th1 and inhibiting the Th2 secretion of IL-4 [61].

The increased expression of IFN-γ following exposure to weaning as a stressor is similar to that seen following an adrenocorticotropic hormone (ACTH) challenge using twelve Brahman heifers [62]. These authors reported that the gene expression of IFN-γ increased 16-fold following ACTH challenge which resulted in an increase in the concentration of endogenous cortisol. Carroll et al. [33] identified increased serum concentration of IFN-γ in weaned beef calves three hours after endotoxin challenge which coincided with the reduction in cortisol concentration from its peak immediately following challenge. However, several recent studies have suggested that the anti-inflammatory effects of glucocorticoids result in decreased expression of pro-inflammatory cytokines, particularly IFN-γ [63-65]. These studies were based on murine models and involved sampling immediately following exposure to a brief physical or psychological stressor (shaking, swimming and electric shock, respectively). In another study, in which LPS was administered beginning 4 days following the use of inescapable tail shock as a stressor, Johnson et al. [66] reported that animals exposed to the stressor had a more potent inflammatory cytokine response within the first hour of LPS challenge than non-stressed controls which is in agreement with the findings of the present study and with those of Burdick et al. [62] and Carroll et al. [33]. It was proposed by Goujon et al. [67] that the *in vivo *pro-inflammatory cytokine response to LPS is inhibited by increased concentrations of glucocorticoids if administered during or immediately following stress, thus resulting in the types of suppression seen in certain studies [63-65]. This theory is further supported by the findings of Carroll et al. [33] in which the pro-inflammatory cytokines IL-1β, IL-6, TNF-α and IFN-γ did not begin to increase until 30 to 120 minutes following the cortisol surge. *In vitro*, studies have shown that long periods of treatment with glucocorticoids are required to suppress IFN-γ signalling beyond several minutes to hours [68]. The findings of Johnson et al. [66], Goujon et al. [67] and Carroll et al. [33] suggest that the increased expression of IFN-γ may be the result of a heightened immune responsiveness following weaning. An earlier sampling time between 1 and 12 h following maternal separation may have resulted in the detection of increased expression of the anti-inflammatory cytokine IL-4.

The expression of a number of adhesion molecules and chemokines is up-regulated by IFN-γ, often synergistically working with IL-1β and TNF-α, to increase margination of lymphocytes and macrophages [60]. The increased expression of IL-1β on days 1, 3 and 7 coincides with the expression of IFN-γ. The concentration of TNF-α remains elevated over a similar time course to IFN-γ in this study, which is in accordance with research indicating that IFN-γ production is stimulated by TNF-α in addition to auto-stimulation [60,69]. Bailey et al. [70] found TNF-α was up-regulated in a murine social stress model, indicating the key role TNF-α plays in stress-induced inflammation. It has also been demonstrated that endogenous cortisol, resembling concentrations present during the physiological stress response, result in markedly increased gene expression of TNF- α in cattle [62].

Expression of TLR4 was up-regulated in all calves following weaning, increasing to an over 2-fold increase on day 7, indicating its potential use as a novel biomarker of weaning stress in the bovine. While TLR4 is well established as a modulator of both innate and adaptive immunity [71], it has also recently been identified as playing a role in non-infectious inflammatory disease [72], with activation of TLR4 increasing the expression of a number of pro-inflammatory cytokines [73]. Zhang et al. [74] demonstrated that TLR4 can be activated by a chronic restraint stressor in mice. These authors were the first to identify stress-induced alterations in TLR4 gene expression, showing a 3-fold increase versus non-stressed controls [74,75]. Detectable levels of stress, based on TLR4 expression, remain at 7 days post weaning, suggesting the adjustment period to weaning induced stress may be longer than the anticipated 7 days.

The mRNA expression of GRα was more than 3-fold increased in all animals on day 1 following maternal separation, validating the work of Burdick et al. [62] which found a peak in endogenous cortisol resulted in the up-regulation of GRα throughout the 4 hour challenge. Two bovine studies found significant down-regulation of GRα within 6 hours of dexamethasone administration [76] or the surge of endogenous cortisol that occurs during parturition [40], but the reliance of these studies on exogenous glucocorticoids or the strikingly different physiological environment of parturition mean they cannot be directly compared with the current study. Buckham Sporer et al. [25,27] reported no significant change in expression of GRα following 9 hours of truck transportation. However, a trend existed whereby GRα expression tended to increase immediately following transportation, an increase that remained at sampling 4.5 h later [25,27]. It is reported that glucocorticoids trigger a number of anti-inflammatory genes and increase neutrophil lifespan by acting on GRα [76]. In turn, the suppression of GRα by abundant glucocorticoid levels is part of the hypothalamic-pituitary-adrenal axis' negative feedback system, preventing unregulated, systemic glucocorticoid induced damage by the immune system [77]. The increase observed in this study and in the study of Buckham Sporer et al. [25] may be due to the reported neutrophilia. GRα is abundantly expressed in neutrophils and they are extremely sensitive to glucocorticoids [40]. It would therefore be reasonable to assume that despite an acute down-regulation of GRα, the increased number of total neutrophils in circulation may have resulted in an increased expression of GRα, despite a decrease in expression on a per cell basis.

The pro-apoptotic gene, Fas, codes for a transmembrane death receptor protein (CD95/APO-1) which is present on a number of cells [78]. Additionally, the cell cycle regulator p21, induced in response to DNA damage, has been shown to be involved in apoptosis with activation following T lymphocyte induced Fas signaling [79,80]. In the current study, Fas increased in expression over 3-fold within 24 hours of weaning contrary to recent work that found expression of Fas to be slightly down-regulated by stress in cattle transported for 9 hours [25], and also in an *in vitro *bovine neutrophil study [81]. The expression of p21 was shown to be over 800-fold increased 4.5 hours into transport induced stress [27], although no alterations in p21 were identified in this study. Nonetheless, Kono et al. [82] demonstrated that apoptosis was actually induced in human T cells that were co-cultured with monocytes from stressed patients. There was no indication of apoptosis in T cells cultured with monocytes from unstressed controls. Additionally, both Fas and p21 were found to be up-regulated (~2-fold) following 2 days of restraint stress in murine models [83,84]. This acceleration in the rate of cellular apoptosis indicates an immunological attempt to restore homeostasis [53]. Increased expression of p21 may be a short lived event and was not found immediately following transport stress by Buckham Sporer et al. [27] despite being highly expressed hours earlier.

The cell adhesion molecule, CD62L, plays an important role in the margination of neutrophils to sites of infection and inflammation [85]. Glucocorticoid induced neutrophilia, similar to what is seen in this study, is generally attributed to the down-regulation of CD62L, causing the de-margination of neutrophils from blood vessels back into circulation [86]. However, despite significant neutrophilia, Buckham Sporer et al. [25] found no alterations in CD62L gene expression in neutrophils over the course of 9 hours of transportation and subsequent monitoring. Significant pharmacological doses of glucocorticoids, as used by Tempelman et al. [54] and Weber et al. [86], may be required to induce this suppression. Lynch et al. [34], who reported that total leukocyte and neutrophil number increased in calves on day 2 after weaning, also found percentage phagocytic neutrophils and mean fluorescence intensity of CD62L positive neutrophils decreased in weaned calves compared with baseline, whereas they were unchanged in control calves. The increase observed in the current study on days 1, 3 and 7 could be due to an increase in the release of mature, CD62L expressing neutrophils from bone marrow, as this has been demonstrated to occur following glucocorticoid exposure [53,86].

## Conclusion

The profoundly increased neutrophil number and N:L ratio, reduced lymphocyte number, alterations to the erythron and an increased expression of pro-inflammatory cytokines leads to the conclusion that weaning stress results in a number of modifications to the immune system which have seemingly enhancive characteristics and serve the purpose of clearing infection at a time of increased exposure to novel pathogens. While stress was traditionally understood to suppress the immune system and result in numerous pathologies [6,87], more recent work suggests that acute stress can actually have salubrious consequences for the immune system, resulting in an enhanced response to pathogenic infection [55], whereas chronic stress suppresses the immune system with resulting changes in immunopathology [52,55,88].

This study has established that a number of robust biomarkers for weaning stress exist. The use of more traditional measurements, including total neutrophil number, lymphocyte number and N:L ratio, combined with the highly sensitive immunogenetic markers, IL-1β, IFN-γ, TLR4, GRα and Fas, provide a framework for investigation in future bovine stress studies.

It would be of interest to characterise the global gene expression response of leukocytes to stress at weaning and future work may serve to elucidate key regulatory genes and pathways. From the present study, it is clear that weaning induced alterations in gene expression may extend beyond the 7 days measured and it is necessary to establish at what point in time this returns to baseline levels. It is also necessary to investigate the effect of combining stressors (weaning, housing, castration, transport) on immunomodulation as an increase in the magnitude of stressors may overwhelm the immune system and result in the kind of chronic or extreme stress that could potentially suppress immunity.

Concerning management at weaning, it is evident that calves, particularly intact male calves, may benefit from a weaning strategy where the calves are allowed contact with the dam but prevented from suckling for a number of days before total separation occurs. However, unless other stressors, such as castration or transportation accompany this event, weaning calves without contact with their dams may have no negative impact on their health and welfare.

## Authors' contributions

BE and MMcG designed the study. AOL, BE and SMW optimized leukocyte isolation. SMW and BE modified the RNA extraction method. AOL and SMW designed primers, optimised protocols for real time RT-qPCR reactions and quality control. AOL and BE performed the experiments. AOL and BE analyzed the data and AOL prepared the manuscript. AOL, BE, MMcG, SMW and SD contributed to, read and approved the final manuscript.
